# Identification of the ternary complex of ribonuclease HI:RNA/DNA hybrid:metal ions by ESI mass spectrometry

**DOI:** 10.1016/j.jbc.2021.100462

**Published:** 2021-02-25

**Authors:** Tomoshige Ando, Nujarin Jongruja, Nobuaki Okumura, Kosuke Morikawa, Shigenori Kanaya, Toshifumi Takao

**Affiliations:** 1 Institute for Protein Research, Osaka University, Osaka, Japan; 2 Graduate School of Engineering, Osaka University, Osaka, Japan; 3 Department of Gene Mechanisms, Graduate School of Biostudies, Kyoto University, Kyoto, Japan

**Keywords:** ribonuclease, metal ion–protein interaction, manganese, zinc, mass spectrometry (MS), DTT, dithiothreitol, EDTA, ethylenediaminetetraacetate, ESI-MS, electrospray ionization–mass spectrometry, HIV-1, human immunodeficiency virus type-1, HPLC, high-performance liquid chromatography, RF, radio-frequency, RT, reverse transcriptase, TEA, Triethylamine

## Abstract

Ribonuclease HI, an endoribonuclease, catalyzes the hydrolysis of the RNA strand of an RNA/DNA hybrid and requires divalent metal ions for its enzymatic activity. However, the mechanistic details of the activity of ribonuclease HI and its interaction with divalent metal ions remain unclear. In this study, we performed real-time monitoring of the enzyme–substrate complex in the presence of divalent metal ions (Mn^2+^ or Zn^2+^) using electrospray ionization–mass spectrometry (ESI-MS). The findings provide clear evidence that the enzymatic activity of the ternary complex requires the binding of two divalent metal ions. The Zn^2+^ ions bind to both the enzyme itself and the enzyme:substrate complex more strongly than Mn^2+^ ions, and gives, in part, the ternary complex, [RNase HI:nicked RNA/DNA hybrid:2Zn^2+^], suggesting that the ternary complex is retained, even after the hydrolysis of the substrate. The collective results presented herein shed new light on the essential role of divalent metal ions in the activity of ribonuclease HI and demonstrate how Zn^2+^ ions confer inhibitory properties on the activity of this enzyme by forming a highly stable complex with the substrate.

Ribonuclease H (RNase H), a ubiquitous enzyme, is found in all organisms from bacteria to mammals (1). RNase H is an endoribonuclease that catalyzes the hydrolysis of the RNA strand of RNA/DNA hybrids and produces 5’-phosphate and 3’-hydroxyl termini. RNase H requires divalent metal ions (Mg^2+^ or Mn^2+^) for its enzymatic activity (2, 3, 4). The important functions of RNases H are: 1) the removal of an RNA strand from an R-loop that is composed of two antiparallel DNA strands plus one RNA strand; 2) removal of RNA fragments (Okazaki fragments) that are produced during DNA replication (5, 6, 7). There are two types of RNase H enzymes; type 1 (RNase HI) and 2 (RNase HII and HIII), the amino acid sequences of which are not conserved except for the active sites, but their tertiary structures resemble each other and have the above activities (8). It is also important to note that RNase HI from *Escherichia coli* (*E. coli*) is quite similar to the C-terminal domain of the reverse transcriptase (RT) of the human immunodeficiency virus type-1 (HIV-1), which actually also has RNase H activity and is considered to be a research target for future therapy (9, 10).

The crystal structures of *E. coli* RNase HI were reported in 1990 by Katayanagi *et al.* (11) and Yang *et al.* (12). Soon afterward, two classes of catalytic mechanisms were proposed to explain the mechanism for the hydrolysis of RNase H, *i.e.*, a one-metal ion mechanism (13, 14) and a two-metal ion mechanism, which was confirmed for the C-terminal domain of RT (15). Despite the structural consistency between RNase HI and the C-terminal domain of RT, the need for more than one divalent metal ion has remained a controversial issue. In 2005, the crystal structure of *Bacillus halodurans* RNase H, which was mutated to an inactive form, complexed with an RNA/DNA hybrid and two Mg^2+^ ions, was reported (16). This finding, as well as other studies (17, 18, 19), provided support for the two-metal ion mechanism, although the structure of a complex composed of native RNase HI, RNA/DNA and a divalent metal ion has never been solved owing to the fact that the substrate promptly undergoes hydrolysis by its own activity, even in the crystalline form. This poses a problem for the structural analysis of a native complex such as an active enzyme and a natural substrate by X-ray crystallography or NMR. In this context, it should be noted that a recent study by time-resolved X-ray crystallography led to the proposal that the canonical two-metal binding mechanism should be revised to a considerable extent (20).

In addition, a higher concentration of metal ion, which is often used in preparing a crystal of a metal-requiring protein, makes the issue of the stoichiometry of the metal ion in the complex controversial (21, 22). In the case of RNase HI, the crystals were prepared using a 100 mM MgSO_4_ solution (23) or 1 mM MnCl_2_ (17) solutions, the concentrations of which were 10 or 100 times higher than those for physiological conditions, respectively. Such high concentrations of metal ions might result in the formation of crystal structures with extra metal ions imbedded within them.

Considering the concentrations of major divalent cations in *E. coli* cells (Mg^2+^: 10^−2^ M, Zn^2+^: 10^−3^ M, Mn^2+^: 10^−5^ M in total), (Mg^2+^: less than 2 mM, Zn^2+^: low nM, Mn^2+^: sub μM in the free form) (24, 25), Mg^2+^ presumably functions as an integral cofactor in the case of RNase HI, which coordinates with the acidic catalytic residues in the active site as demonstrated in its structure (17, 18). Meanwhile, regarding RT, it has been shown that other divalent cations (Zn^2+^ and Mn^2+^) also support the hydrolysis reaction at much lower concentrations, *i.e.*, in the range of a few and several μM, respectively, while these two metal ions could also function as a potent inhibitor of RT in the presence of Mg^2+^ ion (26). The above investigators concluded that the Zn^2+^ inhibition is not due to the inhibition of catalysis but, rather, to the formation of a highly stable, kinetically diminished complex. In fact, RNase H activity is repressed under high concentrations of Mn^2+^ (1 mM) and Mg^2+^ (50 mM) (17). The unique coordination property of several divalent metal ions and the structural commonalty in the active domain of many nucleotide cleaving enzymes, including RT, prompted us to analyze the active complex of RNase HI, an RNA/DNA hybrid, and metal ions, with emphasis on the binding properties of Mn^2+^ and Zn^2+^ as a function of hydrolysis activity.

In recent years, electrospray ionization (ESI) mass spectrometry (MS) has been used for the analysis of native proteins or proteins that are complexed with various compounds. Such measurements can be made on a water-based solvent system, which is close to physiological conditions. This technique has been widely used in the analyses of various types of noncovalent protein–protein and protein–ligand complexes (27, 28, 29, 30, 31). As a result, the stoichiometry of the components in a complex, even those that are transiently formed in solution, can be revealed. In this study, we report, for the first time, the detection of the ternary complex of native RNase HI: RNA/DNA hybrid: divalent metal ions (Mn^2+^ and Zn^2+^). It was possible to monitor a dynamic change in the complex within a few minutes. In addition, the stoichiometry of the Mn^2+^ ion to the RNase HI: substrate was measured by ESI-MS in parallel with an enzymatic activity assay, which allowed us to conclude that the ternary complex, the solution of which showed the most activity, predominantly involves two divalent cations but that a minor fraction is present with only one divalent cation, which essentially supports the canonical “two metal ions mechanism.” Furthermore, the ternary complex containing, in fact, Zn^2+^ ions was found to hold the full-length substrate, but with a “nick” inside. This unexpected observation allowed us to conclude that Zn^2+^ ions are retained within a highly stable complex with an RNA strand, even after the cleavage of the phosphate backbone. This also explains why the properties of the Zn^2+^ ion are different from Mn^2+^ ions in terms of their ability to inhibit RNase H activity *via* the formation of a highly stable complex and also explains the essential role of the second divalent metal ion in hydrolysis activity.

## Results

### RNase HI forms a complex with an RNA/DNA hybrid in the absence of metal ions

**Figure 1:**
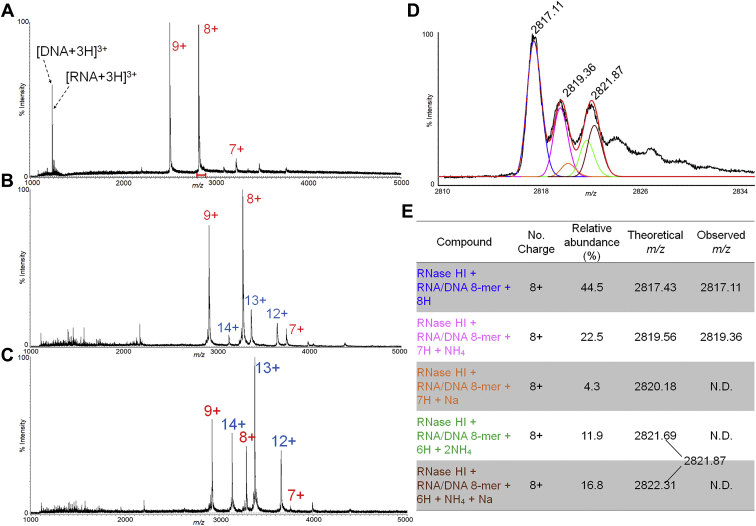
**Detection of RNase HI in complex with an RNA/DNA hybrid in the absence of metal ions by ESI-MS.** ESI mass spectra of a mixture of RNase HI (4 μM) and 8-mer RNA/DNA (4 μM) (*A*), RNase HI (4 μM) and 14-mer RNA/DNA (4 μM) (*B*), and RNase HI (8 μM) and 14-mer RNA/DNA (4 μM) (*C*) in 10 mM NH_4_OAc, pH 7.0. The numbers on the peaks indicate charge states of 1:1 (*red*) and 2:1 (*blue*) complexes of RNase HI and RNA/DNA hybrid. The expanded region of the 8+ charge ion peak is indicated by a *red bar* in *A* (*D*). The major three peaks could be approximately matched with the RNase HI: 8-mer RNA/DNA + 8H+ (*blue*), RNase HI: 8-mer RNA/DNA + 7H^+^ + NH_4_^+^ (*pink*), and a sum of RNase HI: 8-mer RNA/DNA + 6H^+^ + 2NH_4_^+^ (*green*) and RNase HI: 8-mer RNA/DNA + 6H^+^ + NH_4_^+^ + Na^+^ (*brown*) (*E*). Simulative fitting of the observed peaks to the theoretical ones (*red trace*) was carried out by Isotopica, which could concomitantly output the relative abundances of the above ion species (see Experimental procedures).

The ESI-MS of an equimolar mixture of RNase HI and 8-mer RNA/8-mer DNA gave multiply charged ion peaks (7+ to 9+), which corresponded to a 1:1 complex of RNase HI: RNA/DNA hybrid, implying that the complex was stable in solution and the gaseous phase after being sprayed, and its ion particles were maintained during the mass measurement (Fig. 1*A*). Note that the expanded view of the 8+ charged ion showed the adduction of ammonium or/and sodium ions, which are frequently observed in MS, especially, when an aqueous buffer is used. This result indicates that a 1:1 complex could be easily formed without metal ion(s). When the length of the RNA/DNA hybrid was increased to a 14-mer, traces of the 2 (RNase HI): 1 (RNA/DNA) complex were observed (Fig. 1*B*). This 2:1 complex was predominant when the protein concentration was increased to twice that of RNA/DNA hybrid (Fig. 1*C*), implying that a longer RNA/DNA chain would be capable of recruiting multiple enzyme molecules on it. In fact, the crystal structure of the human RNase H C-terminal domain: 14 mer of RNA/DNA hybrid showed that it was a 2:1 complex, in which each RNase H molecule was bound independently to the free space of the RNA/DNA strand (Fig. S1). It is noteworthy that the intensity of the complex with either the DNA and RNA strand was much less than that with the RNA/DNA hybrid (Fig. S2), which could be partly attributed to a lack of synergetic binding force between the strands or either of the strands and the enzyme (see Fig. S1*A*). It should also be noted that only traces of the complex with the single RNA strand were observed (Fig. S2*A*), which can be attributed to the repulsion between 2-OH group of the ribose skeleton and the binding site(s) of the enzyme (19).

### RNase HI shows stoichiometric binding with Zn^2+^, but not with Mn^2+^, Mg^2+^, or Ca^2+^, in the absence of an RNA/DNA hybrid

**Figure 2:**
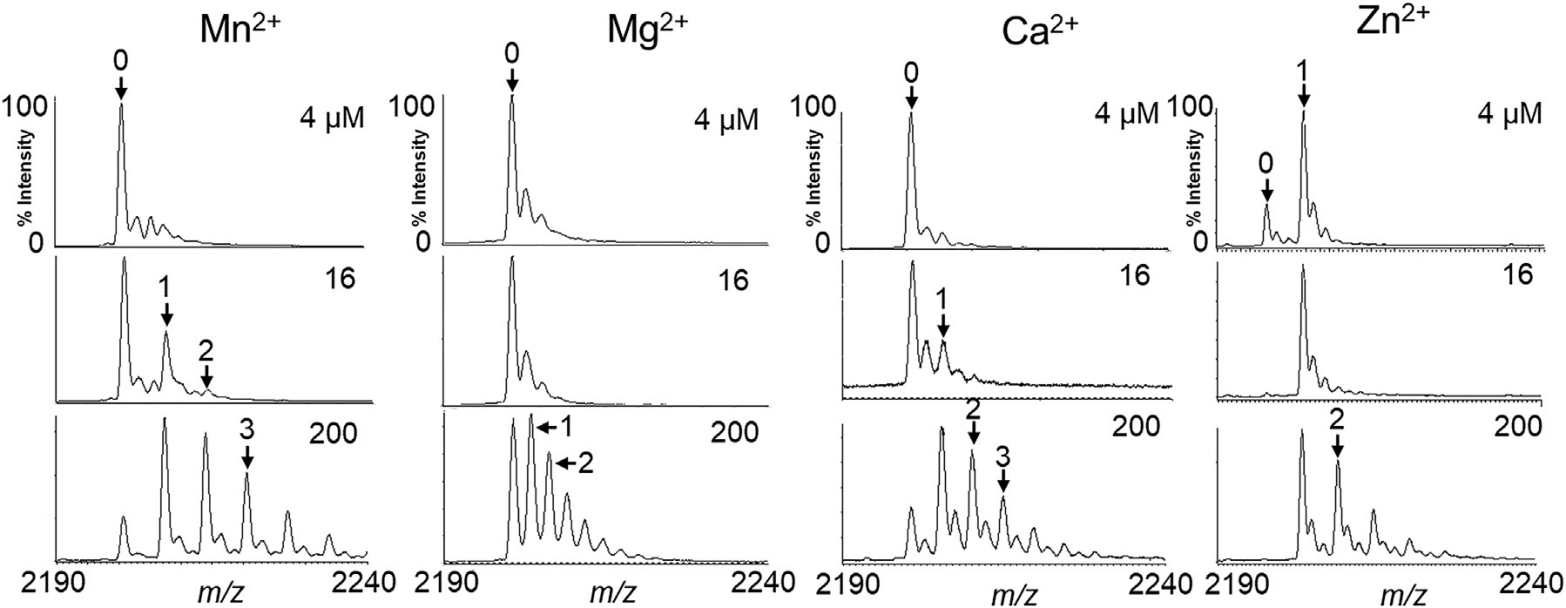
**Binding of divalent metal ions to RNase HI in the absence of an RNA/DNA hybrid.** ESI mass spectra of RNase HI (4 μM) in the presence of 4, 16, and 200 μM of Mn^2+^, Mg^2+^, and Ca^2+^, and 4, 8, and 200 μM of Zn^2+^ in 10 mM NH_4_OAc, pH 6.0. Expanded regions of the 8+ charged ion peaks are shown.

Mg^2+^ at a concentration of a few mM or Mn^2+^ at concentrations of several μM supports optimal enzyme activity (17), while, at RT, Zn^2+^ inhibits the active enzyme with Mg^2+^ at a level of a few μM (26). Analogous to the human RNase H C-terminal domain, the former two metal ions could be coordinated with the carboxylate groups of Asp10, Asp70, Asp134, and Glu48 of RNase HI and a phosphate group of the RNA strand (see Fig. S1), but the structure of the Zn-coordinated enzyme has not yet been solved. Tsunaka *et al.* (32) reported that the binding of the first Mn^2+^ ion eventually triggers the coordination of the two Mn^2+^ ions into the mutant RNase HI using a crystal prepared in the presence of 5 to 10 mM of MnCl_2_. Based on several reports regarding the metal-binding properties of RNase H, it would be interesting to determine whether those divalent metal ions are also specifically bound to the enzyme in solution. In the presence of each metal ion at a concentration of 4 μM, which is stoichiometrically equivalent to the RNase HI molecule, a single Zn^2+^ ion was found to bind to the enzyme in a stoichiometric manner, although this does not necessarily mean that it directly binds to a specific site (Fig. 2). At 16 and 200 μM concentrations of Mn^2+^, Mg^2+^, and Ca^2+^ ions, evidence of metal ion adduction was observed, which is frequently observed in MS of biological compounds and is evidenced by the progressive increase in the number of adduct ions along with increasing concentrations of metal ions. It is possible that these adduct ions could bind nonspecifically to the protein surface by electrostatic interactions.

### The association of Mn^2+^ with RNase HI is required for its RNA-hydrolysis activity

**Figure 3:**
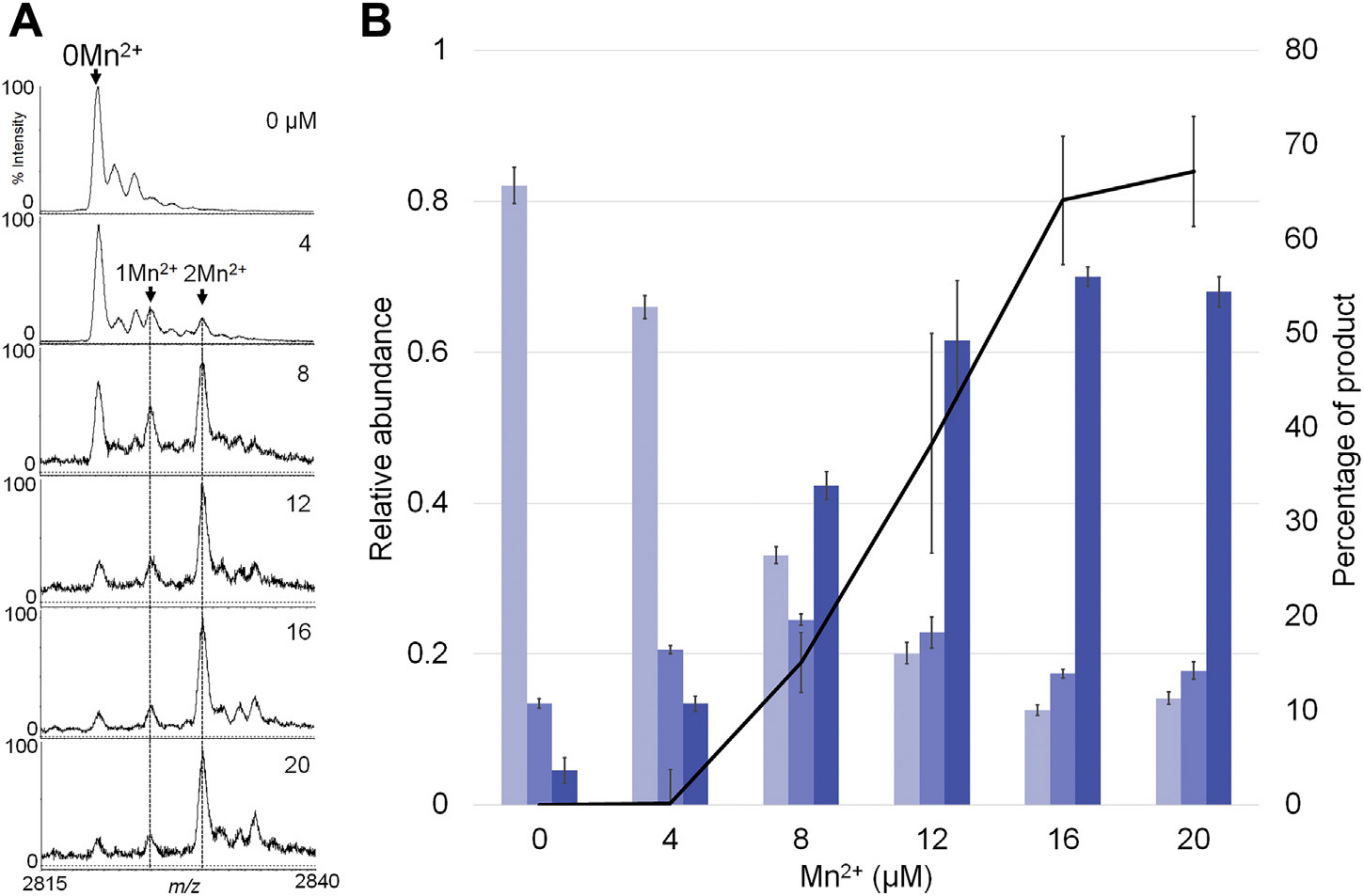
**Metalation states (Mn-form) *versus* enzymatic activity of RNase HI as a function of the amount of Mn**^**2+**^**.** RNase HI (4 μM) and 8-mer RNA/DNA hybrid (10 μM) were incubated in the presence of various concentrations of MnCl_2_ (4, 8, 12, 16, 20 μM) and then subjected to ESI-MS (*A*). The relative intensities of the metalation states (0 Mn^2+^: *light blue*; 1 Mn^2+^: *blue*; 2 Mn^2+^: *thick blue*) were obtained from the average of the 7+ and 8+ charged ion peaks, the latter of which are shown in the *left panel*. In parallel, each enzyme/substrate mixture was separated by LC (see Fig. S3). It gave the “Percentage of product (hydrolysed RNA),” which was calculated by the Equation 1 (*B*). The *bar graphs* were shown as the average of relative intensities of each complex obtained by three independent mass measurements, and the “Percentage of product” was plotted as the average of three independent enzyme activity assays.

In order to detect the ternary complex with enzymatic activity being retained, the solution conditions and measurement parameters for ESI-MS were optimized (see Experimental procedures). Note that the pH was set at 6.0, which was lower than the optimal value (pH 7–8) for expressing activity, because the rate of substrate cleavage under the optimal condition was too fast to permit the enzyme: substrate complex to be detected in the current timescale of mass measurement (∼3.5 min). The enzymatic activity under the above conditions was measured using the 8-mer RNA/8-mer DNA as a substrate, which has a single cleavage site in the RNA molecule, with various concentrations of Mn^2+^ ion (0–20 μM) (Fig. S3). The value was found to be 0.84 pmol (RNA)/min・pmol (RNase HI), based on Equation 1, making it ca. 55 times less active than that obtained under the conditions that were used in Fig. S4. In order to avoid ion suppression in ESI-MS, the concentrations of protein, RNA/DNA hybrid, and divalent metal ion salts were minimized to 4 μM, 10 μM, and 4 to 20 μM, respectively, (Fig. 3). The mass measurement in parallel to the activity assay gave a correlation between complex formation and activity. When the concentration of Mn^2+^ was increased from 4 to 20 μM, which corresponds to 1 to 5 M equivalents to the enzyme, the ternary complex, RNase HI: RNA/DNA: two Mn^2+^, started to be observed at 4 μM of Mn^2+^ and became predominant at a concentration of around 16 μM (Fig. 3*A*), the concentration at which two Mn^2+^ ions could be readily associated with the complex, which was essentially negligible without the substrate (Fig. 2). The relative abundance of the ternary complex with two Mn^2+^ ions (blue bars in Fig. 3*B*) was in good agreement with an increase in enzyme activity. Note that around 70% “Percentage of product” in the activity can be attributed to the short incubation time (2 min) under the limited reaction conditions. When the mixture containing 16 μM Mn^2+^ was allowed to further stand for 3.5 min, the completely hydrolyzed products were obtained (see the inset of Fig. S3). Based on the above observations, it appears that RNase HI requires two Mn^2+^ ions for its activity to be fully expressed.

**Figure 4:**
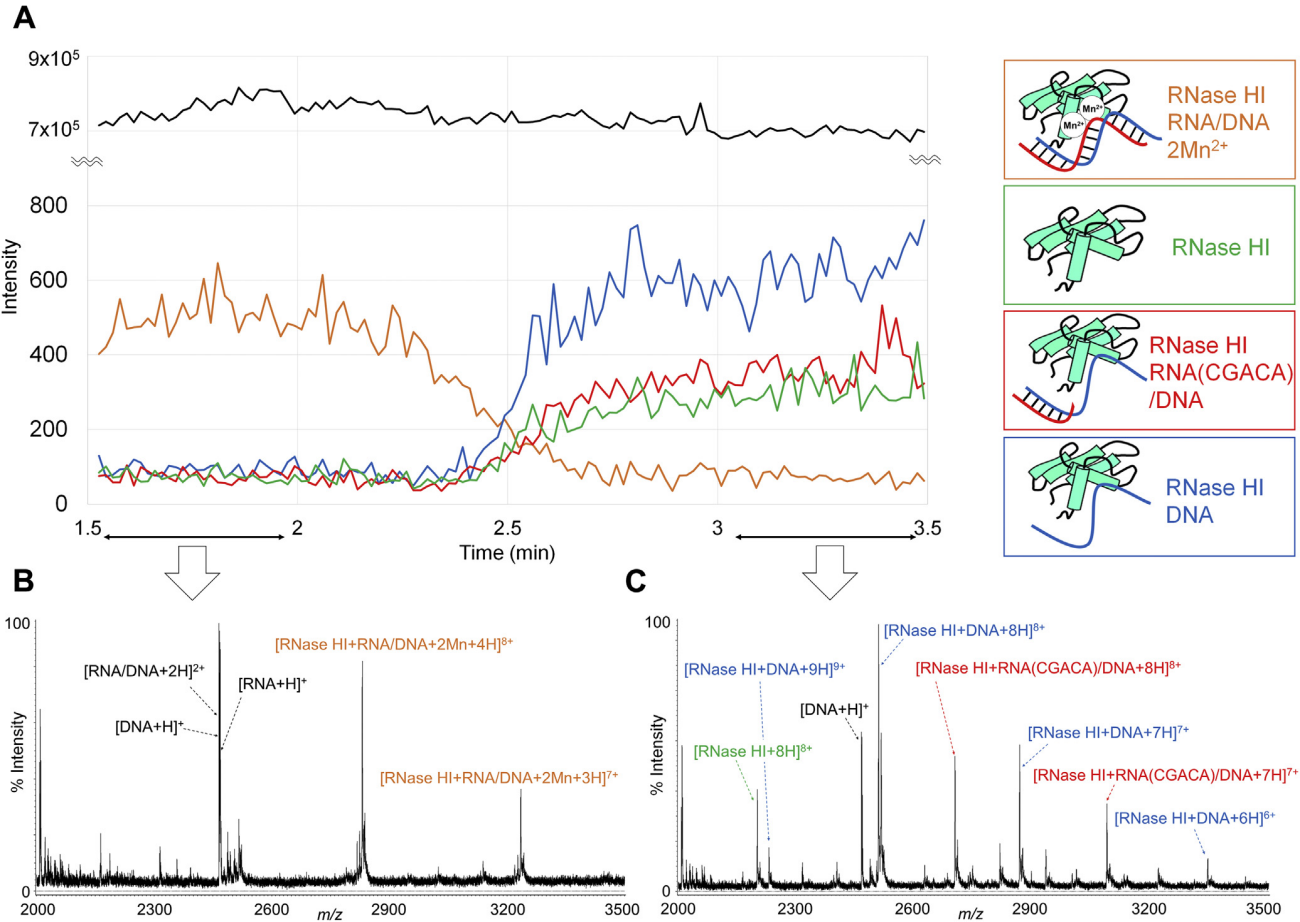
**Real-time monitoring of RNase HI after mixing with an RNA/DNA hybrid in the presence of Mn**^**2+**^**ions.** Total ion chromatogram (*black*) and reconstructed ion chromatograms of RNase HI: 8-mer RNA/8-mer DNA + 4H^+^ + 2Mn^2+^ (*orange*), RNase HI+ 8H^+^ (*green*), RNase HI:8-mer DNA + 8H^+^ (*blue*), and RNase HI: 5-mer RNA/8-mer DNA + 8H^+^ (*red*), which were obtained between 1.5 and 3.5 min (*A*), and mass spectra accumulated between 1.5 and 2 min (*B*) and 3 and 3.5 min (*C*) after mixing RNase HI (4 μM), 8mer RNA/DNA (10 μM), and Mn^2+^ ion (16 μM). The observed m/z values and their assignments are summarized in Table S1.

The preparation with 16 μM Mn^2+^ (four equivalents to the enzyme) was subjected to ESI-MS measurement and continuously monitored from 1.5 to 3.5 min since after mixing all the components (Fig. 4*A*). From 1.5 to 2.0 min, the ternary complex with two Mn^2+^ ions was predominantly observed together with the substrate (Fig. 4*B*); from 2.0 to 3.0 min, the relative abundance of the ternary complex gradually decreased and those of the complexes of RNase HI: 5-mer RNA: 8-mer DNA (red), RNase HI: DNA (blue), and RNase HI (green) correspondingly increased; from 3.0 to 3.5 min, the ternary complex was barely observed, and instead, the latter three components became prominent (Fig. 4*C*). This result indicates that the reaction of the 2.5-fold substrate over the enzyme under the present conditions proceeded rapidly and reached a steady state in around 3 min, none of the resulting products that were comprised of the enzyme itself and that complexed with DNA or DNA/degraded RNA contained a Mn^2+^ ion. The observation of the complex with an intact DNA chain in the final solution provides support for the conclusion that the enzyme favorably binds to the ssDNA chain (Fig. S2). Notably, a minor portion of the RNase HI complex with a single Mn^2+^ was found along with the major complex that contained two Mn^2+^ ions (see Fig. 3). This may be related to the controversial issue of whether a single Mn^2+^ would be mobile within the active site during the progress of the reaction (14).

### The Zn^2+^-bound form of RNase HI holds a nicked RNA/DNA substrate after the hydrolysis reaction is complete

**Figure 5:**
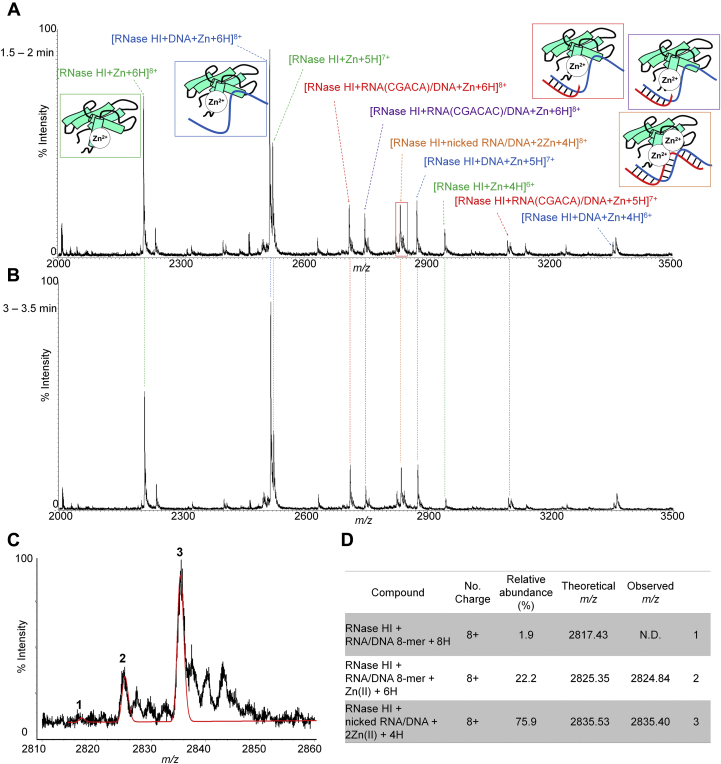
**Snap shots of an RNase HI complex after mixing with an RNA/DNA hybrid in the presence of Zn**^**2+**^**.** ESI mass spectra accumulated between 1.5 and 2 min (*A*), 3 and 3.5 min (*B*) and the expanded region *boxed in red* in *A* (*C*) after mixing RNase HI (4 μM), 8-mer RNA/8-mer DNA (10 μM), and Zn^2+^ ion (16 μM). The observed peaks in *C* could be matched with the two ion species, *i.e.*, RNase HI:RNA/DNA + Zn^2+^ + 6H^+^ and RNase HI: nicked RNA/DNA hybrid + 2Zn^2+^ + 4H^+^ (*D*). The simulative fitting of the peaks to the theoretical ones (*red trace* in *C*) was carried out by Isotopica, which could concomitantly output the relative abundances of each ion species. The observed m/z values in *A* and their assignments are summarized in Table S2.

The ternary complex with a Zn^2+^ ion was tested following the same procedures as above. Unlike the results for the Mn complex, the MS profile remained unchanged throughout the measurement from 1.5 to 3.5 min after mixing the enzyme, substrate, and Zn^2+^ ion (Fig. 5, *A* and *B*). This result indicates that all of the constituents in the solution had already reached a steady state at the start of the measurement, which turned out to be comprised of five reaction products: RNase HI: Zn^2+^ (green), RNase HI: DNA: Zn^2+^ (blue), RNase HI: 5-mer RNA/DNA: Zn^2+^ (red), RNase HI: 6-mer RNA/DNA: Zn^2+^ (violet), and [RNase HI: nicked RNA/DNA hybrid: 2Zn^2+^] (orange). As expected from the results shown in Figure 2, all of the products contained a bound Zn^2+^ ion. The expanded view of the intact ternary complex at the 8+ charge state showed the presence of two ion species at m/z 2824.84 and 2835.40 as the major components, which was in agreement with the calculated values for RNase HI: RNA/DNA: Zn^2+^ (calcd. 2825.35 (8+)) and [RNase HI: RNA/DNA: 2Zn^2+^] +18 (calcd. 2835.53 (8+)), respectively (Fig. 5, *C* and *D*). The latter value was estimated to be the active ternary complex with two Zn^2+^ ions, but to retain the cleaved RNA chain, the +18 Da is due to H_2_O. Such a complex has never been observed for the Mn complex. The above reaction mixture, obtained at the starting point of the mass measurement, was also subjected to high-performance liquid chromatography (HPLC) (see the inset of Fig. S5), supporting the fact that the substrate was completely hydrolyzed at 1.5 min after mixing. This unexpected and surprising finding accounts for the Zn complex forming a stable complex with the RNA chain, even after cleavage had occurred, and further, for the role of the second divalent metal ion for the hydrolysis activity, although the disposition of the Zn^2+^ ions in the structure has not yet been elucidated. It should also be noted that the complex with the 6-mer RNA (colored in violet in Fig. 5*A*) was barely observed for the case of the Mn complex, but the complex with 5-mer RNA, which was the major cleavage product based on the HPLC profile (see Fig. S5), was observed in both cases. This difference in the cleavage products can be attributed to the slight change in the enzyme specificity *via* coordination with Zn^2+^ ions. The result is consistent with a previous report showing that Zn^2+^ ion supports enzyme activity at lower concentrations of Zn^2+^ ion (∼25 μM), and the observation of the complex, [RNase HI: nicked RNA/DNA hybrid: 2Zn^2+^], may account for the inhibitory property of the Zn complex at RT by forming a highly stable, kinetically diminished complex (26).

## Discussion

ESI-MS provided a snapshot of a ternary complex, RNase HI: RNA/DNA hybrid: divalent metal ions (Mn^2+^, Zn^2+^ in this study), which could be transiently formed in buffer solution, and the structure was maintained during the time required to make the MS measurement, which was carried out within a few minutes. The functional relevance of the observed complex was evidenced by the enzymatic activity, which was measured using the same solution that was used in the ESI-MS. With an incremental increase in the Mn^2+^ ion concentration from 0 to 5 mol per mole of RNase HI, the activity reached nearly the maximal level in the present assay conditions (see Experimental procedures), at which the complex comprised of RNase HI: RNA/DNA: Mn^2+^ (1: 1: 2) was observed to be the predominant component. In turn, the level of the RNase HI: RNA/DNA (1: 1) complex was decreased; the RNase HI: RNA/DNA: Mn^2+^ (1: 1: 1) complex was increased to around 8 μM of Mn^2+^, then decreased slightly, but remained in part, even at 20 μM of Mn^2+^ (Fig. 3). This is likely due to the turnover of Mn^2+^ ions during the enzyme reaction. However, considering the fact that the active enzyme with Mg^2+^ was inhibited by 50% in the presence of ∼500 μM MnCl_2_ (26), the two-metal ion form might be product-inhibitory at higher concentrations of Mn^2+^ ion. While the specific affinity of the enzyme for Mn^2+^ ion was nearly negligible, two Mn^2+^ ions apparently coordinated at the same concentration of Mn^2+^ as that in Figure 2 when mixed with an RNA/DNA hybrid (Fig. 3). This can be explained by the fact that the Mn^2+^ ions chelate with the phosphodiester bond at the cleavage site of an RNA chain on one side and with the “DEDD” motif, located in the active site, on the other side (see Fig. S1*B*). In fact, upon the hydrolysis of the RNA chain, this coordination network was cancelled to produce the complexes (see Fig. 4*C*), any of which did not contain Mn^2+^. This highlights the conclusion that the binding of these two Mn^2+^ ions is essentially retained by the cleavage site of the RNA chain.

The “DEDD” motif is conserved not only in RNase H family members but also in various types of exoribonucleases, DNA polymerases, *etc.* (33, 34), and also is known to bind with Zn^2+^ ion (35, 36). In fact, its specific binding to RNase HI was clearly observed (Fig. 2). Moreover, with relatively low concentrations (8 ∼ 16 μM) of Zn^2+^ ion, the RNA chain was cleaved with less than 1.5 min (Fig. 5*A*), while the Mn complex largely persisted for up to ca. 2 min after being mixed with the substrate under the same conditions (Fig. 4*A*). It should also be noted that, while the ternary Mn complex was broken up upon cleavage of the substrate (RNA chain), the ternary Zn complex, [RNase HI: nicked RNA/DNA hybrid: 2Zn^2+^], continued to exist (Fig. 5). This result suggests that the higher affinity of Zn^2+^ ion might render the complex highly stable, which might, in turn, lead to the inhibitory effect of Zn^2+^ ions (26). Such a stable complex with divalent metal ions was also reported for a very short patch repair (Vsr) endonuclease, the ternary structure of which was complexed with duplex DNA containing a TG mismatch, but nicked the phosphate backbone at the active site where the two Mg^2+^ ions were coordinated (37). In both cases, a high affinity of divalent metal ions in the active site could result in product inhibition, *i.e.*, blocking substrate turnover. Real-time monitoring of the ternary complex of native RNase HI, RNA/DNA hybrid, and divalent metal ions demonstrated that two Mn^2+^ ions were specifically associated with the enzyme at a level of 10 μM (Fig. 3) that exhibited the transient activity. However, this does not necessarily indicate that this ternary complex actually functions *in vivo*, taking into consideration the fact that the concentration of Mn^2+^ in *E. coli* is at the sub μM level in the free form. The Mg complex could not be observed by the present method, because of the rapid turnover of Mg^2+^ ions and the need to use a higher concentration (1 mM∼) in order to form the active complex, which severely interfered with the ionization (Fig. S6). Thus, based on these findings, the possibility that the Mg^2+^ ion is the actual player within cells cannot be excluded.

In conclusion, the present study provides direct evidence for the essential role of divalent metal ions (Mn^2+^ and Zn^2+^) for the hydrolytic activity of RNase HI in solution, which overall supports the canonical two-metal ion mechanism for the case of Mn^2+^ ions, meanwhile, Zn^2+^ ions, which have been shown to act as an inhibitor of the RNase H domain of HIV RT (26), was demonstrated to form a stable ternary complex with the substrate even after being hydrolyzed, which provides direct evidence of Zn^2+^ ions being inhibitory. It also highlights the potential of ESI-MS to allow transient complexes to be measured in real time.

## Experimental procedures

### Reagents

Synthetic ssRNA (8-mer: 5’-CGACACCU-3’, 14-mer: 5’-CGACACCUGAUUCC-3’) and ssDNA (8-mer: 5’-AGGTGTCG-3’, 14-mer: 5’-GGAATCAGGTGTCG-3’) were obtained from Biologica Co. A commercial product of recombinant RNase H was from Takara Bio Inc. Ammonium acetate (NH_4_OAc, MS grade), trifluoroacetic acid (TFA), manganese (II) chloride tetrahydrate (99.99%), magnesium chloride hexahydrate (99.995%), zinc chloride (99.999%), calcium chloride, acetic acid, and trizma base were from Sigma-Aldrich. Sodium chloride, dithiothreitol (DTT) and hydrochloric acid were from Wako. Triethylamine (TEA), SDS, tryptone, dried extract yeast, and 0.2 mol/l-di-sodium dihydrogen ethylenediaminetetraacetate (EDTA) solution were from Nacalai Tesque. Ultrapure water was produced by PURIC-ω (ORGANO) and used for all the experiments.

### Expression and purification of RNase HI

*E. coli* RNase HI was expressed in the *rnhA* mutant *E. coli* strain, MIC3009 [*F, supE44, supF58, lacY1 or Δ(lacIZY)6, trpR55, galK2, galT22, metB1, hsdR14 (rK-,mK+), rnh-339: cat*] (38, 39). The *E. coli* cells were transformed with a vector encoding *E. coli* RNase HI (40), inoculated into 2 ml of Luria Bertani (LB) medium containing 50 μg/ml ampicillin, and incubated overnight at 32 °C. The culture was added to 1 l of LB medium with ampicillin (50 μg/ml) and cultivated at 32 °C. When its absorbance at 550 nm became higher than 1.0, the temperature was raised to 42 °C and further cultivated for 4 h. Cells were harvested by centrifugation at 4000 rpm at 4 °C for 5 min and resuspended in 20 ml 10 mM Tris-HCl (pH 7.4) containing 100 mM NaCl, 1 mM EDTA, and 1 mM DTT (TE buffer). The cell suspension was sonicated and centrifuged at 4000 rpm at 4 °C for 15 min. The supernatant was mixed with phosphocellulose equilibrated in TE buffer and stirred by a rotator at 4 °C for 2 h. The resulting slurry was then packed into an empty column, washed with 20 ml TE buffer, and eluted with 10 ml of 10 mM Tris-HCl (pH 7.4) containing 500 mM NaCl, 1 mM EDTA, and 1 mM DTT (39). After being analyzed by SDS-PAGE, RNase HI fractions were collected and concentrated by Amicon Ultra (10 kDa cut, Merck KGaA). The concentrated RNase HI solution (about 50 μl) was further purified by size-exclusion chromatography (see below) and concentrated by Amicon Ultra (10 kDa cut). This purified recombinant enzyme was reconstituted in 25 mM Tris-HCl, pH 7.4, 30 mM NaCl, 0.5 mM EDTA, 1 mM DTT, 50% glycerol, in which the authentic RNase H (Takara Bio Inc) was dissolved. The activity of the resulting enzyme that was obtained was examined by a previously reported method (26) with minor modifications and compared with the authentic one (see Fig. S4).

### Size-exclusion HPLC

Size-exclusion chromatography was carried out using an SEC column (Superdex 75 Increase 5/150 GL, GE Healthcare) in an HP1100 HPLC system (Agilent Technologies, Inc). The column was equilibrated with 25 mM Tris HCl pH7.4, 50 mM NaCl, 1 mM EDTA・2Na, 1 mM DTT, and 20 μl of sample protein was then injected using a manual injector. Proteins were separated at a flow rate of 0.2 ml/min, and RNase HI fractions, monitored by absorbance at 280 nm, were checked by SDS-PAGE.

### Reverse-phase HPLC

The protein concentration of the above final preparation was determined by HPLC using an HP1100 HPLC system. The proteins were separated on a column (Intrada WP-RP 1.0 × 150 mm, 3 μm, Imtakt Co) that had been equilibrated with 95% solvent A (0.1% TFA-H_2_O)/5% solvent B (0.1% TFA-ACN) and eluted using a linear gradient of solvent B (5–80%) in 40 min at a flow rate of 80 μl/min. The peak area at 280 nm of RNase HI, which had been quantified by amino acid analysis, was used for estimating the protein amount.

### Preparation of RNA/DNA hybrids

Synthetic single-strand (ss) RNA and ssDNA (8 and 14 mers for each), dissolved in distilled water at a concentration of 1 nmol/μl, were purified in an HP1100 HPLC system using a C_18_ reversed-phase column (COSMOSIL 5C_18_-PAQ, 4.6 × 150 mm, 5 μm, Nacalai Tesque). Both ssRNA and ssDNA were separated using a linear gradient 5 to 80% in 40 min of solvent B (25% ACN in 0.075 M TEA-acetic acid (TEAA) buffer, pH 7.0) in solvent A (0.1 M TEAA buffer, pH 7.0) at a flow rate of 1 ml/min. The purified ssRNA (8-mer and 14-mer) and ssDNA (8-mer and 14-mer) were dissolved in 10 mM NH_4_OAc pH 7.0, and then, equimolar mixtures, *i.e.*, RNA/DNA hybrids, were prepared on the basis of the concentrations obtained from the absorption at 260 nm.

### Electrospray MS

ESI-MS was carried out using AccuTOF JMS-T100LC mass spectrometer (JEOL). To optimize the transmission of high-m/z protein complex ions, the radio-frequency (RF) power supply for the quadrupole-type ion guide, situated between the atmospheric pressure ionization interface and the TOF mass analyzer, was modified to lower the RF from the standard 3 MHz to 0.8 MHz, and the pressure of the first differential pumping region was increased from about 200 Pa to 320 Pa. ESI was achieved using a glass nanospray tip (HUMANIX). The tip was positioned manually using an in-house prepared grabber. All samples were dissolved in a 10 mM NH_4_OAc solution (pH 6.0 for all the mass measurements except for those in Fig. 1, which were obtained with the same buffer but at pH 7.0) and introduced manually into the nanospray tip. Mass measurements were carried out by applying a voltage of +1680 V to the nanospray tip in the positive ion mode. The flow rate of the drying gas (N_2_) was set at 30 l/min, and the source temperature behind the first skimmer was 40 °C. RNase HI and mixtures thereof with ssRNA, ssDNA, or the RNA/DNA hybrid or/and divalent metal ions (MgCl_2_, CaCl_2_, ZnCl_2_, or MnCl_2_) were prepared prior to mass measurements. Real-time reaction monitoring was started at 1.5 min after mixing the above solutions. During this 1.5 min interval, the mixture was introduced into the tip and then placed in the ESI source, where data were continuously acquired for up to 3.5 min at which time, the ternary complex was barely observed. All the data were analyzed by Mass Center software (JEOL). The relative ratios of the components involved in the peak envelops were analyzed by Isotopica (http://coco.protein.osaka-u.ac.jp/isotopica/) (41).

### Enzyme activity assay

The enzyme activity was determined as follows: The enzyme (40 pmol) and the 8-mer RNA/8-mer DNA hybrid (100 pmol) were dissolved in 10 μl of 10 mM NH_4_OAc (pH 6.0) containing 0, 4, 8, 12, 16, and 20 μM MnCl_2_, and each solution was allowed to stand for 2 min at 32 °C, the conditions of which were set so as to allow for mass measurements (see Results). The reaction mixtures were then quenched by adding 10 μl of a 0.1% TFA solution, containing 1 mM EDTA, and separated by reverse-phase HPLC. The same mixture solutions as the above, but without quenching, were immediately subjected to ESI-MS. RNA, the fragmented RNA, and DNA could be separated on a column (Cadenza CD-C18, 1.0 × 150 mm, 3 μm, Imtakt Co) at a flow rate of 0.08 ml/min using the same buffer system as was used for the preparation of RNA/DNA hybrids with the linear gradient of buffer B (0–5 min: 5–30%; 5–30 min: 30–60%; 30–31 min: 60–80%; 31–36.1 min: 80%; 36.1–50 min: 5%). The peak areas of the original RNA (8-mer), observed at 260 nm, were normalized by the area ratio of the DNA peak, obtained in each chromatogram, to that obtained in the absence of Mn^2+^ ion, since the DNA strand was resistant to enzyme cleavage. The enzyme activity was estimated, based on the decrease in the intensity of the RNA peak from that obtained without Mn^2+^ ion (Equation 1). This assay was repeated three times, averaged, and plotted as a function of the amount of Mn^2+^.

(1) $$\text{Percentage}\;\text{of}\;\text{product}\;\left(\text{cleaved}\;\text{RNA}\right)=100\;\text{X}\;\left[\text{R0}-\frac{\left[\text{R}\right]}{\frac{\left[\text{D}\right]}{\left[\text{D0}\right]}}\right]/\text{R0}$$

R and D denote 8-mer RNA and 8-mer DNA peak areas obtained at each concentration of Mn^2+^; R0 and D0 denote peak areas obtained without Mn^2+^.

## Data availability

Any data that support the findings of this study are included within the article and its supplementary information files.

## Supporting information

This article contains supporting information.

## Conflict of interest

The authors declare no conflicts of interest in regard to this article.
