# Supplementary material for: Identification of the ternary complex of ribonuclease HI:RNA/DNA hybrid:metal ions by ESI mass spectrometry
Source: J Biol Chem. 2021 Feb 25;296:100462. doi: 10.1016/j.jbc.2021.100462 (PMC8042393; doi:10.1016/j.jbc.2021.100462)
Supplement: Supplemental Figures S1–S6 and Tables S1–S2 [file mmc1.pdf]

Supporting Information

Identification of the ternary complex of ribonuclease HI:RNA/DNA hybrid:metal ions by ESI mass spectrometry

Tomoshige Ando[1†], Nujarin Jongruja[2], Nobuaki Okumura[1], Kosuke Morikawa[3], Shigenori Kanaya[2], and Toshifumi Takao[1*]

[1]Institute for Protein Research, Osaka University, Osaka, Japan

[2]Graduate School of Engineering, Osaka University, Osaka, Japan

[3]Department of Gene Mechanisms, Graduate School of Biostudies, Kyoto University, Kyoto, Japan

[†]Present Address: Center for Instrumental Analysis, Kyoto Pharmaceutical University, Kyoto, Japan

*Corresponding author: Toshifumi Takao

E–mail: tak@protein.osaka-u.ac.jp

Running title: Real-time monitoring of active ternary complex of RNase HI

Keywords: ribonuclease, metal ion ‐ protein interaction, manganese, zinc, mass spectrometry (MS)

CONTENTS

Figure S1. The crystal structure of the complex of human RNase H C-terminal domain: RNA/DNA hybrid: Ca^{2+} ions.

Figure S2. ESI mass spectra of equivalent mixtures of RNase HI and 8-mer RNA, and RNase HI and 8-mer DNA.

Figure S3. HPLC profiles of the products generated by the action of RNase HI (4 μM) in the presence of Mn^{2+} ion at 0, 4, 8, 12, 16, 20 μM.

Figure S4. Comparison of HPLC profiles of the products (substrate) generated by recombinant *E. coli* RNase HI prepared in-house and commercial recombinant RNase H.

Figure S5. HPLC profiles of the products generated by the action of RNase HI (4 μM) in the presence of Zn^{2+} ion at 0, 4, 8, 12, 16, 20 μM.

Figure S6. HPLC profiles of the products generated by the action of RNase HI (4 μM) in the presence of Mg^{2+} ions at 0, 16, 32, 100, 1,000, 10,000 μM (A) and ESI mass spectra of the above reaction mixtures with Mg^{2+} ions at 200 and 1,000 μM (B).

Table S1. Summary of observed and theoretical mass values and their corresponding compounds in Figure 4.

Table S2. Summary of observed and theoretical mass values and their corresponding compounds in Figure 5.

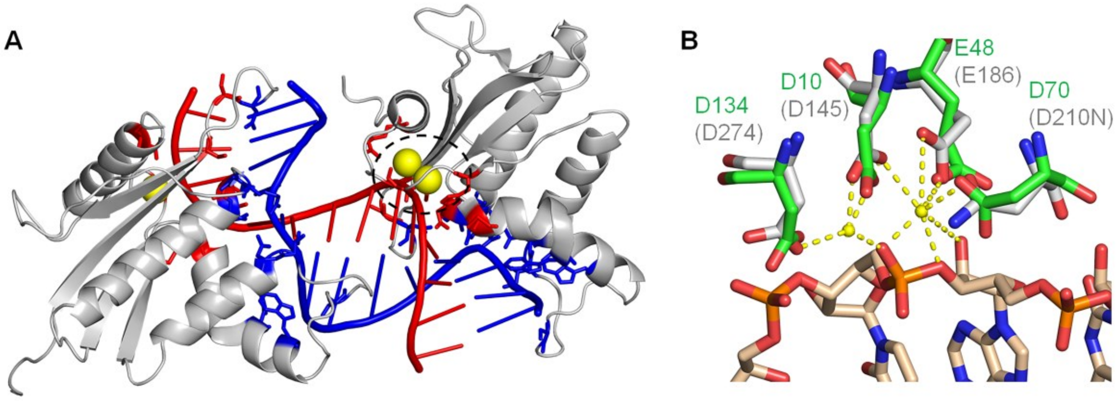

Figure S1. The crystal structure of the complex of the human RNase H C-terminal domain (gray): RNA (red)/DNA (blue) hybrid: Ca^{2+} ions (yellow sphere) (PDB ID; 2QKK) [1]. RNA and DNA binding sites were shown by red and blue sticks, respectively (A). Expanded view of the catalytic site of human RNase H, circled in broken line in A, which is mutated (D210→N) and complexed with the RNA strand (colored in ivory) by the electrostatic interaction via two Ca^{2+} ions (yellow sphere) (B). The green sticks (D10, E48, D70, D134), derived from *E. coli* RNase HI (PDB ID; 1RDD) [2], are overlaid on human RNase H.

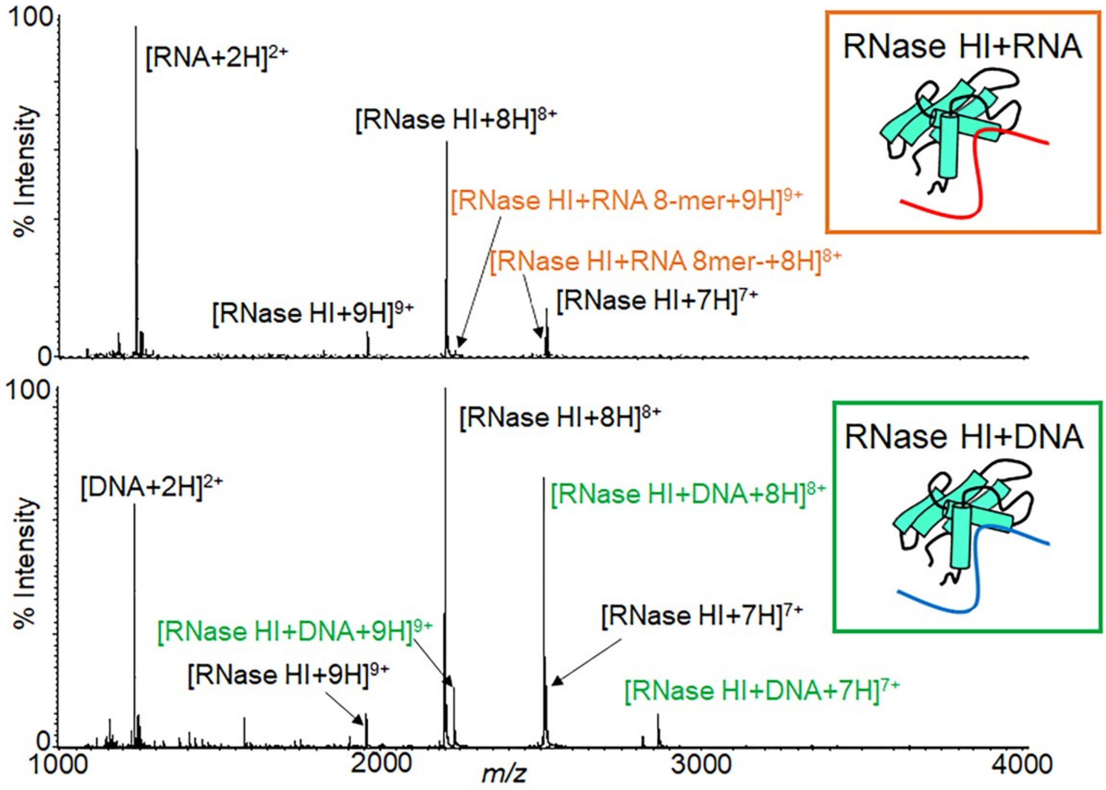

Figure S2. ESI mass spectra of equimolar mixtures (4 μM) of RNase HI and 8-mer RNA (A), and RNase HI and 8-mer DNA (B) in 10 mM NH_4OAc, pH 6.0.

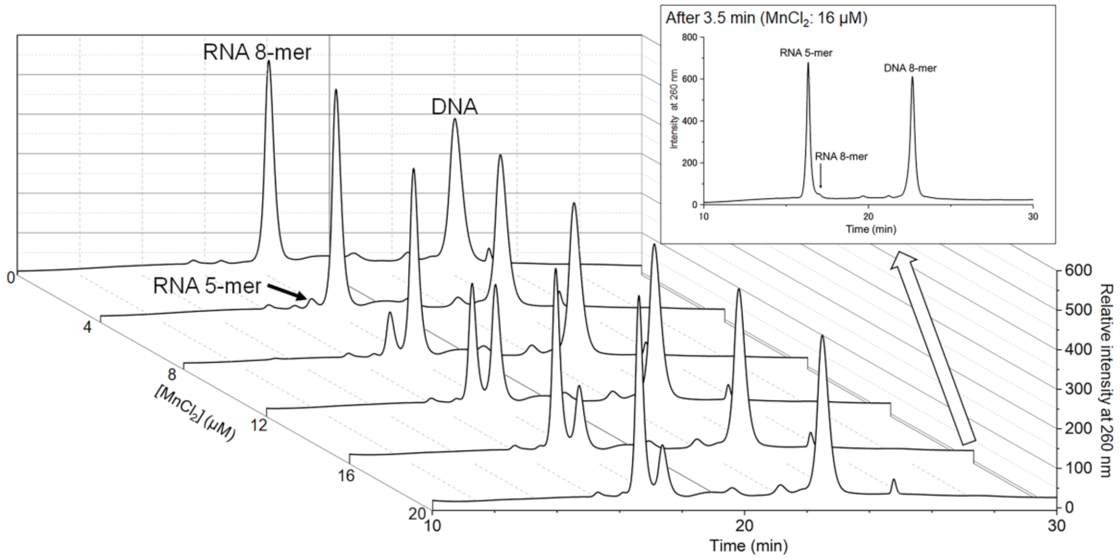

Figure S3. HPLC profiles of the products generated by the action of RNase HI (4 μM) in the presence of Mn^{2+} ion at 0, 4, 8, 12, 16, 20 μM. The peaks of the original RNA (8-mer) and fragmented RNA (5-mer) were normalized to the DNA peak obtained without Mn^{2+} ion. 8-mer RNA (substrate): CGACACCU; 5-mer RNA (product): CGACA; 8-mer DNA: AGGTGTCG. RNase HI (40 pmol) and the 8-mer RNA/DNA hybrid (100 pmol) were dissolved in 10 μL of 10 mM NH_4OAc (pH 6.0) containing 0, 4, 8, 12, 16, and 20 μM $MnCl_2$, and each solution was allowed to stand for 2 min at 32 °C, and the solution containing 16 μM $MnCl_2$ was further stood for 3.5 min (inset). The reaction was quenched by 10 μL of a 0.1% TFA solution containing 1 mM EDTA, and half of the solution (10 μL) was injected into reverse-phase HPLC (see "Enzyme Activity Assay" in MATERIAL and METHODS).

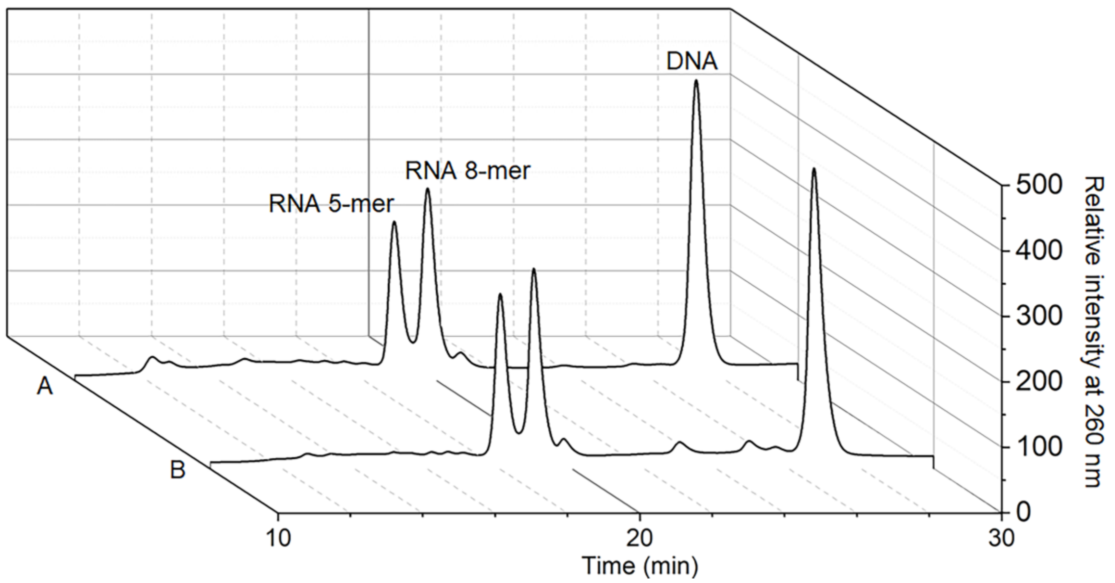

Figure S4. Comparison of HPLC profiles of the products (substrate) generated by recombinant *E. coli* RNase HI prepared in-house (A) and commercial recombinant RNase H (B). 8-mer RNA (substrate): CGACACCU; 5-mer RNA (product): CGACA; 8-mer DNA: AGGTGTCG. RNase HI or commercial RNase H (0.5 pmol for each) and the 8-mer RNA/DNA hybrid (100 pmol) were dissolved in 10 μL of 25 mM Tris-HCl (pH 7.4) containing 20 μM $MnCl_2$ and 50 mM KCl, and incubated for 2 min at 37 °C. The reaction was quenched by adding 10 μL of a 0.1% TFA solution containing 1 mM EDTA, and half of the solution (10 μL) was injected to reverse-phase HPLC (see "Enzyme Activity Assay" in MATERIAL and METHODS).

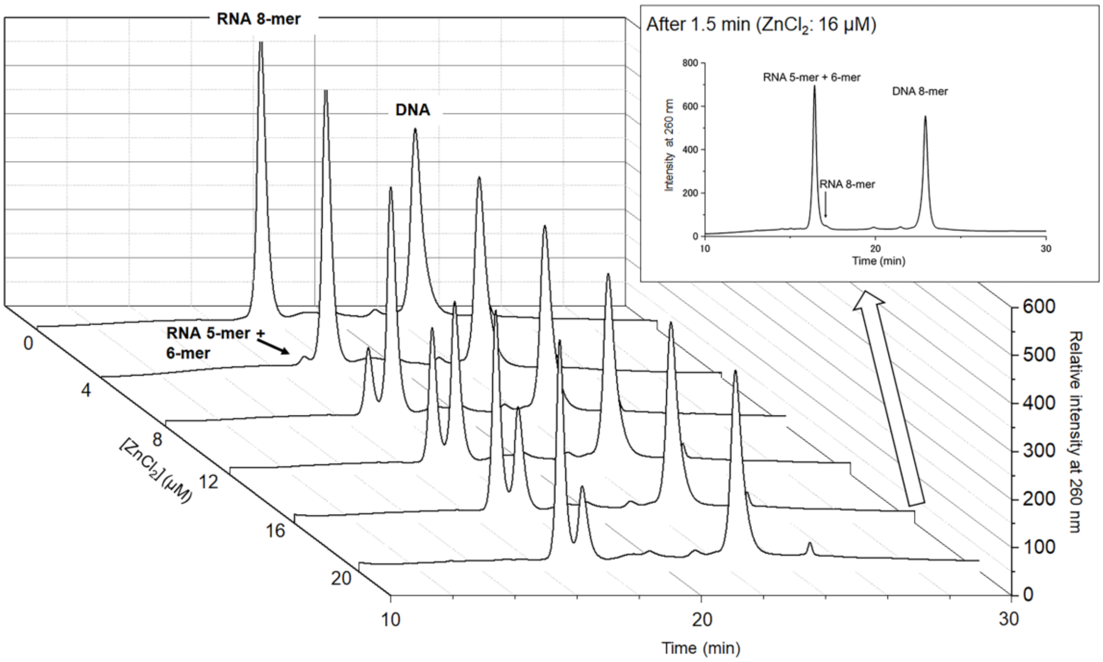

Figure S5. HPLC profiles of the products generated by the action of RNase HI (4 μM) in the presence of Zn^{2+} ion at 0, 4, 8, 12, 16, 20 μM. The peaks of the original RNA (8-mer) and fragmented RNA (5-mer and 6-mer) were normalized to the DNA peak obtained without a Zn^{2+} ion. 8-mer RNA (substrate): CGACACCU; 5-mer RNA (product): CGACA; 6-mer RNA (product): CGACAC; 8-mer DNA: AGGTGTCG. 5-mer RNA and 6-mer RNA were eluted at the same retention time under the present HPLC conditions. RNase HI (40 pmol) and the 8-mer RNA/DNA hybrid (100 pmol) were dissolved in 10 μL of 10 mM NH_4OAc (pH 6.0) containing 0, 4, 8, 12, 16, and 20 μM $ZnCl_2$, and each solution was allowed to stand for 20 sec at 32 °C. The reaction was quenched by adding 10 μL of a 0.1% TFA solution containing 1 mM EDTA, and half of the solution (10 μL) was injected into reverse-phase HPLC. The same reaction mixture as used in Fig. 5 in the text was also subjected to HPLC (inset).

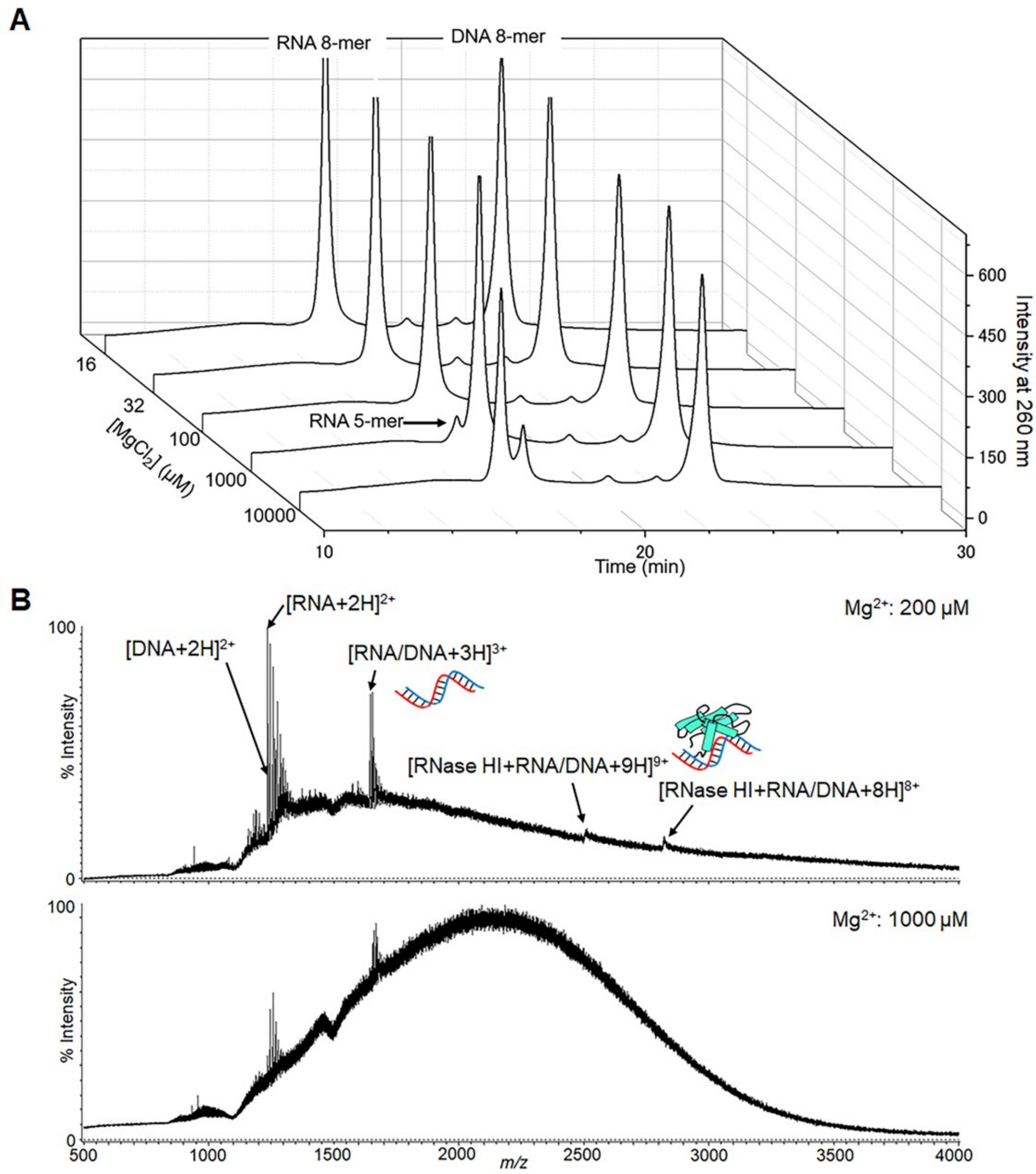

Figure S6. (A) HPLC profiles of the products generated by the action of RNase HI (4 μM) in the presence of Mg^{2+} ions at 0, 16, 32, 100, 1,000, 10,000 μM. RNase HI (40 pmol) and the 8-mer RNA/DNA hybrid (100 pmol) were dissolved in 10 μL of 10 mM NH_4OAc (pH 6.0) containing 0, 16, 32, 100, 1,000 and 10,000 μM $MgCl_2$ and each solution was allowed to stand for 1 min at 32 °C. The reaction was quenched by adding 10 μL of a 0.1% TFA solution containing 1 mM EDTA, and half of the solution (10 μL) was injected into the reverse-phase HPLC. (B) ESI mass spectra of the above reaction mixtures with Mg^{2+} ions at 200 μM (upper) and 1,000 μM (lower) before quenching. [RNase HI + RNA/DNA + 9H or 8H]$^{9+\text{ or }8+}$ denote the enzyme-substrate (non-cleaved) complex. No cleaved RNA products were detected in both spectra.

Table S1. Summary of observed and theoretical mass values and their corresponding compounds in Figure 4.

No.	Compound	Number of Charge	Theoretical m/z	Observed m/z
1	DNA + H^+	1	2465.46	2465.52
2	RNA + H^+	1	2468.39	2468.47
3	RNA/DNA + $2H^+$	2	2466.93	2466.52
4	RNase HI + RNA/DNA + $2Mn^{2+}$ + $4H^+$	8	2830.66	2830.70
5	RNase HI + RNA/DNA + $2Mn^{2+}$ + $3H^+$	7	3234.90	3234.95
6	RNase HI + $8H^+$	8	2200.65	2200.74
7	RNase HI + $7H^+$	7	2514.88	2515.12
8	RNase HI + DNA + $9H^+$	9	2230.21	2230.25
9	RNase HI + DNA + $8H^+$	8	2508.86	2508.90
10	RNase HI + DNA + $7H^+$	7	2867.12	2867.20
11	RNase HI + DNA + $6H^+$	6	3344.81	3344.95
12	RNase HI + RNA(CGACA)/DNA + $8H^+$	8	2702.86	2702.92
13	RNase HI + RNA(CGACA)/DNA + $7H^+$	7	3088.84	3088.96

Table S2. Summary of observed and theoretical mass values and their corresponding compounds in Figure 5.

No.	Compound	Number of Charge	Theoretical m/z	Observed m/z
1	RNase HI + Zn^{2+} + $6H^+$	8	2208.57	2208.52
2	RNase HI + Zn^{2+} + $5H^+$	7	2523.94	2523.95
3	RNase HI + Zn^{2+} + $4H^+$	6	2944.43	2944.38
4	RNase HI + DNA + Zn^{2+} + $6H^+$	8	2515.78	2516.75
5	RNase HI + DNA + Zn^{2+} + $5H^+$	7	2876.18	2876.08
6	RNase HI + DNA + Zn^{2+} + $4H^+$	6	3355.37	3355.36
7	RNase HI + RNA(CGACA)/DNA + Zn^{2+} + $6H^+$	8	2710.79	2710.70
8	RNase HI + RNA(CGACA)/DNA + Zn^{2+} + $5H^+$	7	3097.90	3097.88
9	RNase HI + RNA(CGACAC)/DNA + Zn^{2+} + $6H^+$	8	2748.93	2748.89
10	RNase HI + RNA/DNA + $2Zn^{2+}$ + H_2O + $4H^+$	8	2835.53	2835.46

References

1. Nowotny, M., Gaidamakov, S. A., Ghirlando, R., Cerritelli, S. M., Crouch,R. J., and Yang, W. (2007) Structure of human RNase H1 complexed with an RNA/DNA hybrid: Insight into HIV reverse transcription. *Mol. Cell*, **28**, 264–276
2. Katayanagi, K., Okumura, M., and Morikawa, K. (1993) Crystal structure of *Escherichia coli* RNase HI in complex with Mg^{2+} at 2.8 Å resolution:Proof for a single Mg^{2+}-binding site. *Proteins*, **17**, 337–346
